# Cardiovascular Risks in Relation to Daidzein Metabolizing Phenotypes among Chinese Postmenopausal Women

**DOI:** 10.1371/journal.pone.0087861

**Published:** 2014-02-12

**Authors:** Zhao-min Liu, Suzanne C. Ho, Yu-ming Chen, Jun Liu, Jean Woo

**Affiliations:** 1 Department of Medicine &Therapeutics, the Chinese University of Hong Kong, Hong Kong SAR, China; 2 Division of Epidemiology, The Jockey Club School of Public Health and Primary Care, the Chinese University of Hong Kong, Hong Kong SAR, China; 3 Department of Medical Statistics and Epidemiology, School of Public Health, Sun Yat-sen University, Guangzhou, PR, China; Universidad de Valladolid, Spain

## Abstract

**Background:**

Studies suggested that the inter-individual differences in metabolizing isoflavone daidzein to equol or O-desmethylangolensin (ODMA) might explain the inconsistency of the soy/isoflavones efficacy on cardiovascular health.

**Objectives:**

The study aims to evaluate the relationship between equol and ODMA phenotypes and cardiovascular risks with habitual isoflavone consumption in Chinese postmenopausal women.

**Methods:**

This is a cross-sectional study among 726 prehypertensive postmenopal women who were screened for a randomized controlled trial. 648 women returned a daidzein-challenged urine samples for determination of equol and O-DMA production. 595 attended clinic visits for assessment of cardiovascular risks including body composition, blood pressure (BP), serum lipids, uric acid, high sensitivity C-reactive protein (hs-CRP), fasting glucose and free fatty acid (FFA).

**Results:**

The prevalences of equol and O-DMA producers were 53.2% and 60.9% respectively. Equol producers had higher fat free mass (p = 0.001), lower systolic (p = 0.01) and diastolic (p = 0.01) BP, serum triglyceride (p = 0.023), hs-CRP (p = 0.015) and FFA (p = 0.001) than non-producers. O-DMA producers had lower body fat% (p = 0.032), SBP (p = 0.02), total cholesterol (p = 0.002) than non-producers. The significant differences remained after further adjustment for potential confounders. The habitual soy isoflavones intake had little relation to cardiovascular risk factors in either equol/O-DMA producer phenotypes.

**Conclusion:**

Equol/O-DMA producers had more favorable cardiovascular risk profiles than non-producers in prehypertensive postmenopausal women.

## Introduction

Soy foods are rich sources of isoflavones, including daidzein and genistein, and are traditionally consumed by Asian populations. Several large-scale prospective cohort studies show that higher soy/isoflavones consumption is associated with favorable cardiovascular health [1]–[3]. However, human intervention studies assessing the effects of soy or isoflavone on cardiovascular risks have reported inconsistent findings [4]–[6]. Studies suggested that the inter-individual differences in gut bacteria metabolism of isoflavone daidzein to equol or *O*-DMA might explain these discrepancies [7]. The daidzein-metabolizing phenotypes appear to remain stable within an individual over time [8], suggesting that physiologic effects of these phenotypes could have long-term impact on the health of human host.

In vitro and animal experiments indicated that equol is more biologically active than its precursor daidzein with a longer half life, greater antioxidant and estrogenic activities [9]. Studies have suggested that the clinical effectiveness of soy/isoflavones might be due to individual’s ability to produce equol in the gut [10]. *O*-DMA is less structurally similar to 17β-estradiol than daidzein and may exhibit different biological actions from daidzein [11]. Evidence from in vitro studies suggests that O-DMA has several cancer-related biological actions [11]. However, human studies examining the relationship of O-DMA phenotypes to health outcomes are limited. Factors that influence the capacity to produce equol/O-DMA are not clearly established; however, gut physiology, host genetics, and diet factors are probably related to the individual difference of equol or ODMA producing [12]. Studies on the association of equol and ODMA phenotypes with cardiovascular risk factors are inconsistent and limited [13], [14]. One study^16^ among 202 Chinese man and women aged 20–69 years reported that equol excretors showed significantly lower serum triglyceride and Common Carotid Intima-Media Thickness (CCA-IMT) than non-excretors. Another study by Liu et al. [14] suggested that habitual isoflavone intake has no significant effect on serum lipids in healthy participants, regardless of equol phenotype. The inconsistency could be due to different population features, methods used to define equol- and ODMA-producers, the small sample size, or the risk parameters observed.

In most of previous reports, daidzein-metabolizing phenotypes were not determined in the context of a standardized phenotyping method. Studies specifically conducted among postmenopausal women investigating the association of equol and O-DMA production and cardiovascular risk are limited [11]. Therefore, the present study aims to assess the association of daidzein metabolizing phenotypes with cardiovascular risks (body composition, blood pressure and cardiovascular biomarkers) among Hong Kong Chinese postmenopausal women who are usual soy consumers. We hypothesize that equol or O-DMA producers have more favorable profiles in cardiovascular health than non-producers. The study would provide important information on the explanation of the inconsistency of the soy efficacy in clinical trial and provide important evidence for future interventions.

## Materials and Methods

### Study Participants

This is a cross-sectional study. Participants were recruited from women who were screened for participation in a six-month randomized controlled trial; a study designed to examine the effect of whole soy and purified daidzien on blood pressure and other cardiovascular risks among Hong Kong postmenopausal women with prehypertension and untreated early hypertension. We screened 726 postmenopausal women recruited from the community through advertisement on newspaper from April to Dec, 2011. Their eligibility was initially assessed with a pretested screening questionnaire through telephone interview. The study was conducted according to the guidelines of Declaration of Helsinki. The study protocol has been approved by the ethical committee of CUHK and written informed consent has been obtained from all participants.

### Subject Recruitment and 24 h Urine Collection

Women after prescreening interview were invited to the health center for an introduction talk, and training for 24 h urine collection. They were given a labeled and graduated urine collection bag and asked to collect 24 h urine on the last day after a consecutive 7 days of 60 mg daidzein challenge. Women who were taking acute antibiotics therapy were re-scheduled for urine collection after 2 weeks since cessation of antibiotic therapy. Urine samples were stored in sealed 4*5-mL tubes at −85°C less than 2 weeks until analysis for isoflavonoids [equol, daidzein, genistein, ODMA, and dihydrodaidzein (DHD)] by HPLC methods. Subjects who have donated 24 h urine samples were further invited for a clinic visit for blood taking, anthropometric measurements and questionnaire interview.

### Inclusion and Exclusion Criteria

Subjects were Hong Kong Chinese women aged 48–70 y; at least 1 year after the cessation of menstruation; with mean SBP 120∼180 mmHg or DBP 80∼100 mmHg or both, based on an average of total 4∼6 resting BP readings in 2 different days. Equol-producer was defined according to a standard method as 24-hour urinary log_10_ S-equol/daidzein ratio greater than –1.75 after daidzein challenge [15]. *O*-DMA-producers were defined as individuals with any detectable concentration of *O*-DMA. Daidzein concentrations <100 µg/L are considered indicative of noncompliance [16].

Subjects were excluded if they were on anti-hypertensive medication, hormone therapy or hypoglycemic agents in recent 3 months; medical history or presence of certain chronic diseases such as stroke, cardiac infarction, severe liver and renal diseases; breast or uterine or ovarian cancer or other malignancies, or abnormal uterine bleeding in recent 5 years; known soy allergy and chronic antibiotic therapy (>4 weeks). More details regarding subjects recruitment were published elsewhere [17].

### Data Collection

Individual information was collected by trained interviewers by face-to-face interview based on pretested and validated questionnaires on socio-demographic data, years since menopause, medical history, medication, dietary habits and physical activities [18]. Information about habitual dietary intakes within the past 12 months was evaluated by a validated food frequency questionnaire (FFQ) [19]. Subjects received a 30-min training on estimation of food amounts, portion and utensil sizes. Food models were used to help subjects in the quantification of their diet. The FFQ includes 89 foods items that cover the most commonly consumed foods in Hong Kong, China. For each food item or food group, subjects were asked about the frequency (daily, weekly, monthly, annually or never) and the amount in small, moderate or large bowl. The energy adjusted nutrients intake (energy, protein, total fat, carbohydrate and soy isoflavones) was calculated based on the China Food Composition Table [20]. Habitual physical activities were assessed by modified Baecke questionnaire validated in Hong Kong population [18].

Anthropometric measurements were collected for body weight, height, waist and hip circumferences according to standard protocols. Body mass index (BMI) and waist to hip ratio (WHR) were calculated. Blood pressure was measured according to standard methods using a validated oscillometric technique (Omron M4-I Intellisense, Omron Corporation, Japan). Body fat percentage (BF%), fat mass (FM) and fat free mass (FFM) were determined by bioelectrical impedance analyzer (BIA, TBF-410-GS Tanita Body Composition Analyzer, Japan). The coefficient of variation for repeated measures was less than 5% for body fat percentage.

Overnight fasting (10–12 h) venous blood samples and 24-h urine samples were obtained. Blood withdrawal for participants with acute inflammation or taking anti-inflammation drugs (i.e. aspirin or antibiotics) was postponed 2 week after cease of treatment. Serum was centrifuged at 3000 g for 15 min at 4°C and isolated within 2 h after collection. Each subject’s serum samples was divided into several aliquots and stored at −85°C until analysis. Serum biochemical analyses were performed on Hitachi 7101 automated analyzer (Japan) at a certified clinical lab. Serum fasting glucose, total cholesterol (TC) and triglycerides (TG) were measured by standardized enzymatic colorimetric methods (HUMAN Dignostics, Germany). Serum HDL-C and LDL-C were measured by enzymatic clearance assay (Daiichi Pure Chemicals Co., ltd, Tokyo, Japan). High sensitive C-reactive protein (Hs-CRP) was tested by particle enhanced immuno-turbidimetry method (Orion Diagnostica Oy, Finland). Serum free fatty acid (FFA) was determined by colorimetric methods (DiaSys, Germany). The intra and inter assay coefficients of variations (CV) of above bio-parameters were 1.3% and 2.3% for glucose, 2.2% and 4.3% for TC, 4.8% and 9.1% for TG, 1.5% and 3.4% for HDL-C, 1.8% and 3.9% for LDL-C, 5.4% and 9.7% for hs-CRP, and 2.9% and 4.9% for FFA.

The daidzein, genistein, equol and O-DMA were assayed using an improved High-performance liquid chromatography (HPLC) methods [21]. Urine samples were extracted by ethyl acetate after deconjugation by β-glucuronidase/sulphatase. After reduced pressure drying, the extract was reconstituted in mobile phase solution for analysis. The HPLC system consisted of a C_18_ stationary phase extraction (5 µm, 4·6ø×250 mm) column, and separation of isoflavones was achieved by gradient elution with the mobile phase of 20–70% methanol at a flow rate of 1·0 ml/min. Isoflavones were detected from the UV absorbance at 254 nm and 280 nm. The standards of daidzein, genistein, equol, and β-glucuronidase were purchased from Sigma Chemical Company (St. Louis, MO, USA). The intra assay CVs for isoflavonoids in the quality control sample, measured in duplicate for each batch, were <10%. The inter assay CVs were <15%.

### Statistical Power

Assuming a 50% rate of equol producers in a total sample of 648 participants who donated 24 hr urine samples, we would have 99% power to find a net difference of 5 mmHg in SBP (SD 9.0 mmHg), and 3 mmHg DBP (SD 6.0 mmHg), and 0.5 mmol/l in total cholesterol (SD 0.9 mmol/l) between euqol producers and non-producers, based on a conventional assumption of α level 0.05 (for a 2-side t-test).

### Data Analysis

Data were checked for normality, and skewed parameters were log transformed before statistical analysis and presented as arithmetic means and standard deviations. Differences between producers and non-producers of equol and ODMA were assessed using t-tests for continuous variables and chi square tests for categorical variables. General linear models (GLM) were used to compare the cardiovascular markers between equol/O-DMA producer and non-producer after adjustment for potential confounding factors. Adjustment variables were those significantly associated with cardiovascular markers and equol or ODMA-producer status in univariate analyses or based on prior knowledge. We also compared the cardiovascular markers (body composition, blood pressure and serum biochemical markers) in low and high isoflavones consumers by equol/ODMA phenotypes by GLM models. Because of its skewed distribution, isoflavone intake was divided into 2 groups (high and low isoflavone intake) by using median intake. All analyses were conducted with SPSS Windows (version 16.0; SPSS, Inc.) and the significant level was set at 0.05.

## Results

Of the 726 women who attended the introduction talk, 648 returned a validly daidzein-challenged urine samples. 595 attended the clinic visits for blood drawing, anthropometric measurements and questionnaire survey, of which 573 completed the food frequency questionnaires (FFQ). The mean age (SD) of these participants was 57.8 (4.7) years with average 8.5 years after menopause.

Equol/ODMA producers had notably higher urinary equol/ODMA execration than non-producers (**Table 1**). The prevalences of producers of equol, *O-*DMA and both were 53.2%, 60.9% and 32.6% respectively. No significant association between the two phenotypes was observed (*χ* ^2^ = 0.048, *p* = 0.826). There were no significant difference between equol/O-DMA producers and non-producers in terms of demographic and reproductive characteristics such as age, menopausal years, age at menarche, ever use of hormones replace treatment or contraceptives, total physical activity (PA), habitual tea, alcohol and coffee drinking etc (**Table 2**). Women with different daidzein-metabolizing phenotypes had no significant differences in dietary intakes of protein, total fat, carbohydrate, dietary fiber and isoflavones. However, equol producers were more likely to intake less energy (P = 0.044), and both equol and ODMA producers took more sports PA than non-producers (p = 0.000).

**Table 1:** Urinary isoflavones levels after isoflavone daidzein challenge (µmol/24 hours).

	Non-equol producers	Equol-producers	P	Non-O-DMA producers	O-DMA producers	P
n (persons)	292	305		256	341	
DHA	3.96±6.02	3.61±2.29	0.352	4.20±6.15	3.47±2.67	0.051
Daidzein	9.25±7.01	11.07±17.99	0.107	9.43±18.54	10.75±8.63	0.250
Glycitein	0.48±1.00*	0.86±1.21	0.000	0.57±1.03	0.76±1.19	0.041
Equol	0.02±0.26*	3.66±3.12	0.000	1.95±2.32	1.82±3.24	0.566
Genistein	2.07±1.67	2.22±1.82	0.288	1.99±1.45	2.26±1.93	0.064
O-DMA	1.48±1.38	1.35±1.39	0.248	0.00±0.00*	2.48±0.85	0.000

Data are presented as mean ± standard deviation. DHA denotes dihydrodaidzein; O-DMA denotes O-desmethylangolensin;

*denotes significant difference between producers and non-producers by t-test (P<0.05).

**Table 2:** Demographic, reproductive and lifestyle characteristics of participants by equol and O-DMA phenotypes.

	Non-Equol producers		equol producers	P	Non-O-DMA producers	O-DMA producers	P
Demographic and reproductive characteristics							
n		292	305		256	341	
Age (y)		57.6±4.6	57.9±4.8	0.527	58.1±5.0	57.6±4.5	0.208
Menopausal years (y)		8.2±5.3	8.8±5.9	0.217	8.8±6.2	8.4±5.3	0.365
Age at menarche(y)		13.2±1.8	13.0±1.8	0.380	13.3±1.8	13.0±1.8	0.090
Ever use of contraceptives(%)		126 (50.6)	154 (46.7)	0.191	98 (45.2)	182 (50.3)	0.264
Ever use of HRT (%)		38 (15.3)	51 (15.5)	0.522	36 (16.6)	53 (14.6)	0.303
**Dietary intake (from FFQ)**							
n		244	329		213	360	
Energy (kcal/d)		2100.7±1018.6*	1958.0±912.2	0.044	2031.7±1013.6	2011.2±929.4	0.805
Protein (g/1000 kcal)		55.8±13.2	54.9±13.5	0.419	54.5±13.5	55.8±13.3	0.274
Total fat (g/1000 kcal)		21.2±6.9	20.9±6.8	0.612	21.1±6.5	20.9±7.0	0.752
Carbohydrate (g/1000 kcal)		166.3±17.0	167.0±17.6	0.598	166.5±16.0	166.8±18.1	0.865
Fiber (g/1000 kcal)		21.0±8.8	20.4±8.3	0.389	20.2±8.2	20.9±8.6	0.292
Cholesterol (mg/1000 kcal)		146.2±90.1	146.9±77.4	0.292	143.8±78.4	148.3±85.7	0.538
Isoflavones (mg/1000 kcal) #		7.03±7.83	6.12±6.84	0.145	7.05±8.44	6.37±6.19	0.263
**Other lifestyle factors**							
N		292	305		256	341	
Total PA (MET-min/d)		1445.6±728.2	1413.0±816.1	0.620	1460.4±808.8	1407.0±761.0	0.427
Occupational PA		586.1±759.5	474.4±741.1	0.077	502.2±731.8	534.8±762.2	0.615
Housework PA		558.7±459.9	549.8±489.5	0.823	555.9±446.6	552.2±494.3	0.929
Sports PA		141.0±166.2*	219.0±254.8	0.000	169.9±192.3*	211.4±267.9	0.031
Sitting hours (h/d)		3.3±1.9	3.3±2.1	0.981	3.2±2.1	3.4±2.0	0.273
Regular tea drinking(%)		195 (78.6)	268 (81.7)	0.397	176 (82.2)	287 (79.3)	0.447
Regular alcohol drinking(%)		18 (7.3)	30 (9.1)	0.256	19 (8.8)	29 (8.0)	0.757
Regular coffee drinking(%)		84 (33.9)	127 (38.7)	0.256	78 (36.4)	133 (36.7)	0.944
Smoking (%)		1 (0.4)	2 (0.6)	0.672	1 (0.5)	2 (0.6)	0.693
Passive smoking (%)		43 (17.4)	48 (14.6)	0.211	37 (15.3)	54 (15.0)	0.481

Data are presented as mean ± standard deviation for continuous variables or number (%) for categorical variables. T-test was applied for continuous variables and Chi-square test for categorical variables. HRT indicates hormone replacement treatment; FFQ indicates food frequency questionnaires; PA indicates physical activity; Regular drinking means drinking alcohol, tea or coffee more than 1 time per week; METs are multiples of resting metabolic rates and a MET-minute is computed by multiplying the MET score of an activity by the minutes performed. Dietary nutrients intakes were calculated mainly based on the China Food Composition Table 2002 and 2004.

# denotes testing by non-parametric Mann-Whitney Test due to data heterogeneity;

*denotes significant difference between producers and non-producers by t-test (P<0.05).

For the variables of body composition (**Table 3**), in both unadjusted and adjusted models, equol producers had higher fat free mass than non-producers, while O-DMA producers tent to have lower BMI and less body fat% than non-producers. No differences were observed in body weight, waist circumference, WHR between producers and non-producers within either equol or ODMA phenotypes.

**Table 3:** Body composition, blood pressure and other cardiovascular biomarkers in relation to equol-producer and *O*-DMA-producer phenotypes.

		Non-equol producers	Equol producers	P unadjusted	P’ adjusted	Non-O-DMA producers	O-DMA producers	P unadjusted	P’ adjusted
		(n = 292)	(n = 305)			(n = 256)	(n = 341)		
**Body composition**									
Body weight (kg)		55.7±9.0	56.0±8.8	0.374	0.272	56.6±8.9	55.9±8.9	0.319	0.119
BMI (kg/m^2^)		23.0±3.2	23.3±3.3	0.602	0.471	23. 6±3.3#	23.1±3.3	0.072	0.022
WC (cm)		77.5±8.8	78.1±8.3	0.378	0.310	78.1±8.1	77.7±8.6	0.325	0.304
HC (cm)		92.5±6.8	93.1±6.9	0.262	0.352	93.1±6.8	92.7±6.8	0.469	0.290
WHR		0.838±0.050	0.838±0.047	0.730	0.251	0.840±0.040	0.837±0.051	0.811	0.581
Body fat%		29.3±6.8	30.0±6.4	0.091	0.103	30.6±6.6* #	29.4±6.6	0.032	0.023
Fat mass (kg)		17.8±6.5	17.6±6.6	0.306	0.410	18.2±7.1	17.5±7.2	0.257	0.175
Fat free mass (kg)		37.5±4.7* #	38.7±3.8	0.001	0.001	38.0±4.4	38.3±4.2	0.334	0.866
**Blood pressure**									
SBP (mmHg)		135.1±16.6* #	131.6±16.0	0.010	0.015	135.8±16.8* #	131.5±15.9	0.020	0.010
DBP (mmHg)		83.1±9.6* #	79.4±8.7	0.010	0.010	81.7±9.2	80.6±9.3	0.171	0.338
**Other CVD biomarkers**									
FG (mmol/L)	5.41±0.76		5.38±0.88	0.626	0.186	5.44±0.90	5.36±0.78	0.254	0.355
TG (mmol/L)	1.41±0.79*		1.30±0.63	0.023	0.191	1.37±0.76	1.33±0.68	0.517	0.886
TC (mmol/L)	5.76±0.90		5.63±0.94	0.089	0.423	5.84±0.92* #	5.59±0.91	0.002	0.002
HDL-C (mmol/L)	1.72±0.39		1.71±0.41	0.700	0.371	1.71±0.35	1.74±0.47	0.100	0.158
LDL-C (mmol/L)	3.66±0.97		3.55±0.84	0.169	0.348	3.68±0.84#	3.55±0.93	0.056	0.049
Hs-CRP (mg/L)	1.90±2.08* #		1.61±1.90	0.015	0.021	1.78±2.00	1.71±1.98	0.500	0.219
Uric acid (µmol/L)	289.2±79.5		289.4±67.6	0.982	0.581	283.1±77.8	293.0±69.8	0.050	0.422
FFA (µmol/l)	612.9±204.6* #		554.5±202.6	0.001	0.002	592.7±199.9	572.0±208.4	0.153	0.237

Data are presented as mean ± standard deviation. Skewed variables or variables with heterogeneity in variance (serum glucose, triglyceride, total cholesterol and high sensitivity C-reactive protein) were corrected by log transformation and reported arithmetic means± standard deviation. T-test was applied for continuous variables. BMI indicates body mass index; WC indicates waist circumference; HC indicates hip circumference; WHR indicates waist to hip ratio; DBP and SBP indicates diastolic and systolic blood pressure; FG indicates fasting glucose; TC indicates total cholesterol; TG indicates triglycerides; HDL-C indicates HDL-cholesterol; LDL-C indicates LDL-cholesterol; hs-CRP indicates high-sensitivity C-reactive protein; FFA indicates free fatty acid;

*indicates significant difference between producers and non-producers by t-test (unadjusted P<0.05).

# indicates significant difference between producers and non-producers by general linear model (GLM) (adjusted P<0.05); P’ adjusted is derived from GLM by adjustment for potential confounders. For markers of body composition and blood pressure, the adjusted variables include age, menopausal year, sports physical activity, dietary energy, fat and isoflavones; For other CVD biomarkers, the adjusted variables include age, menopausal years, sports physical activity, dietary energy, fat, isoflavones and BMI.

For the variables of other cardiovascular markers (**Table 3**), in unadjusted analyses, equol producers had lower systolic (p = 0.01) and diastolic (p = 0.01) BP, serum triglyceride (p = 0.023), hs-CRP (p = 0.015) and free fatty acid (p = 0.001) than equol non-producers. ODMA producers had lower SBP (p = 0.02), total cholesterol (p = 0.002) and LDL-c (p = 0.056) than non-producers. The differences remained statistically significant after further adjustment for age, menopausal years, sports PA, dietary energy and lipids, and body fat free mass.

### Effects of Soy-isoflavone Consumption on Cardiovascular Markers, by Equol/ODMA Phenotypes

The median habitual isoflavones intake among the participants was 4.7 mg/1000 kcal/d (0.9∼17.3 mg/1000 kcal), which was defined as the cut-off value between low and high isoflavone levels. Interaction tests were performed by GLM analysis before subgroup analyses. There were marginal or significant interactions between equol producing status and habitual isoflavones intake in triglycerides (p = 0.047) and hs-CRP (p = 0.056) levels; ODMA status and isoflavones intake in triglycerides (p = 0.021) and total cholesterol (p = 0.059) levels. As shown in **Table 4** and **Table 5**, after adjustment of potential confounders, equol producers with high habitual isoflavones intake had lower triglycerides level than non-producers (p = 0.018), and equol non-producers had lower hs-CRP level (p = 0.044) with higher isoflavones intake.While in non-ODMA producers, higher habitual isoflavones intake was associated with lower triglycerides (p = 0.002) and total cholesterol (p = 0.026) levels. No significant differences were found for other biomarkers between low and high habitual isoflavones groups in either equol or ODMA phenotypes.

**Table 4:** Comparison of body composition, blood pressure and serum biochemical markers in low and high isoflavones consumers by equol phenotypes.

	Non-equol producers		P	Equol- producers		P
	Low isoflavonesintake (n = 114)	High isoflavonesintake (n = 128)		Low isoflavonesintake (n = 169)	High isoflavonesintake (n = 158)	
	2.9 mg/1000 kcal	10.3 mg/1000 kcal		2.9 mg/1000 kcal	10.3 mg/1000 kcal	
**Body composition**						
BMI (kg/m^2^)	22.8±3.2	23.5±3.4	0.604	23.22±2.95	23.40±3.71	0.636
WC (cm)	76.9±8.8	78.4±8.5	0.612	77.84±8.06	78.47±9.14	0.399
HC (cm)	91.8±6.8	93.1±6.7	0.491	92.85±6.34	93.48±7.66	0.215
WHR	0.84±0.05	0.84±0.05	0.920	0.84±0.05	0.84±0.05	0.886
Body fat%	28.8±6.7	30.0±6.7	0.131	30.2±6.0	30.4±7.1	0.485
Fat mass (kg)	17.4±7.8	18.6±7.9	0.633	17.13±5.60	18.13±7.89	0.109
Fat free mass (kg)	37.3±4.3	37.6±4.9	0.963	38.43±3.55	38.81±4.06	0.235
**Blood pressure**						
SBP (mmHg)	132.4±17.4	133.1±17.6	0.808	127.1±16.9	125.8±17.5	0.215
DBP (mmHg)	82.4±11.1	83.5±10.2	0.280	76.2±9.7	76.0±10.2	0.473
**Other CVD biomarkers**						
FG (mmol/L)	5.41±0.61	5.45±0.81	0.973	5.34±0.84	5.41±0.75	0.442
TG (mmol/L)	1.39±0.80	1.48±0.75	0.255	1.38±0.55*	1.26±0.69	0.018
TC (mmol/L)	5.68±1.01	5.84±0.86	0.252	5.61±0.92	5.69±0.97	0.478
HDL-C (mmol/L)	1.76±0.41	1.79±0.39	0.810	1.70±0.43	1.72±0.34	0.475
LDL-C (mmol/L)	3.59±0.97	3.80±1.04	0.189	3.54±0.79	3.61±0.91	0.735
Hs-CRP (mg/L)	2.15±2.39*	1.74±1.73	0.044	1.55±1.80	1.79±2.17	0.259
Uric acid (µmol/L)	303.2±82.6	286.2±77.5	0.112	285.8±67.8	295.4±65.4	0.341
FFA (µmol/l)	577.0±184.5	642.6±217.5	0.052	563.3±204.9	548.8±208.7	0.220

Data are presented as mean ± standard deviation. Skewed variables or variables with heterogeneity in variance (body fat%, serum glucose, triglyceride, total cholesterol and high sensitivity C-reactive protein) were corrected by log transformation and reported arithmetic means± standard deviation. BMI indicates body mass index; WC indicates waist circumference; HC indicates hip circumference; WHR indicates waist to hip ratio; DBP and SBP indicates diastolic and systolic blood pressure; FG indicates fasting glucose; TC indicates total cholesterol; TG indicates triglycerides; HDL-C indicates HDL-cholesterol; LDL-C indicates LDL-cholesterol; hs-CRP indicates high-sensitivity C-reactive protein; FFA indicates free fatty acid;

*indicates significant difference between high and low isoflavones intake groups by general linear model (GLM) (P<0.05)by adjustment for potential confounders. For markers of body composition and blood pressure, the adjusted variables include age, menopausal year, sports physical activity, dietary energy, fat, isoflavones and ODMA producing status; For other CVD biomarkers, the adjusted variables include age, menopausal years, sports physical activity, dietary energy, fat, isoflavones, BMI and ODMA producing status.

**Table 5:** Comparison of body composition, blood pressure and serum biochemical markers in low and high isoflavones consumers by ODMA phenotypes.

		Non-ODMA producers			ODMA- producers		
		Low isoflavones intake(n = 107)	High isoflavones intake (n = 112)	P	Low isoflavones intake (n = 172 )	High isoflavones intake (n = 167)	P
		2. 9 mg/1000 kcal	10.3 mg/1000 kcal		2. 9 mg/1000 kcal	10.3 mg/1000 kcal	
**Body composition**							
Body weight (kg)		55.80±7.61	55.33±8.41	0.108	56.14±9.14	56.76±9.28	0.380
BMI (kg/m^2^)		23.32±2.90	22.91±3.16	0.228	23.11±3.41	23.65±3.39	0.572
WC (cm)		77.30±7.41	77.62±8.94	0.078	77.91±8.58	78.38±8.40	0.678
HC (cm)		92.80±6.31	92.21±6.69	0.375	93.18±6.97	93.21±7.05	0.167
WHR		0.83±0.05	0.84±0.05	0.034	0.83±0.05	0.84±0.05	0.398
Body fat%		30.42±5.64	29.11±6.73	0.693	29.74±6.74	30.74±6.51	0.366
Fat mass (kg)		17.20±5.51	17.28±7.10	0.060	17.81±7.56	18.23±7.17	0.495
Fat free mass (kg)		37.97±3.87	37.97±3.93	0.419	38.37±4.12	38.07±4.52	0.342
**Blood pressure**							
SBP (mmHg)		129.5±18.1	131.4±17.5	0.744	129.0±16.9	127.2±17.8	0.211
DBP (mmHg)		77.6±10.5	80.3±10.9	0.252	79.2±10.7	78.4±10.5	0.313
**Other CVD biomarkers**							
FG (mmol/L)	5.32±0.72		5.39±0.78	0.028	5.35±0.71	5.44±0.79	0.523
TG (mmol/L)	1.23±0.70*		1.35±0.75	0.002	1.38±0.65	1.36±0.68	0.441
TC (mmol/L)	5.66±0.89*		5.62±0.99	0.026	5.59±0.87	5.83±0.93	0.825
HDL-C (mmol/L)	1.86±0.37		1.68±0.34	0.473	1.68±0.33	1.83±0.46	0.842
LDL-C (mmol/L)	3.54±0.79		3.57±0.89	0.083	3.58±1.02	3.69±0.84	0.957
Hs-CRP (mg/L)	1.89±2.32		1.67±1.85	0.907	1.83±2.01	1.76±2.16	0.796
Uric acid (µmol/L)	283.5±75.7		297.7±71.9	0.879	294.0±65.2	285.4±76.8	0.641
FFA (µmol/l)	575.3±201.8		563.8±194.0	0.513	578.0±228.8	593.2±201.4	0.852

Data are presented as mean ± standard deviation. Skewed variables or variables with heterogeneity in variance (body fat%, serum glucose, triglyceride, total cholesterol and high sensitivity C-reactive protein) were corrected by log transformation and reported arithmetic means± standard deviation. BMI indicates body mass index; WC indicates waist circumference; HC indicates hip circumference; WHR indicates waist to hip ratio; DBP and SBP indicates diastolic and systolic blood pressure; FG indicates fasting glucose; TC indicates total cholesterol; TG indicates triglycerides; HDL-C indicates HDL-cholesterol; LDL-C indicates LDL-cholesterol; hs-CRP indicates high-sensitivity C-reactive protein; FFA indicates free fatty acid;

*indicates significant difference between high and low isoflavones intake groups by general linear model (GLM) by adjustment for potential confounders (P<0.05). For markers of body composition and blood pressure, the adjusted variables include age, menopausal year, sports physical activity, dietary energy, fat, isoflavones and equol producing status; For other CVD biomarkers, the adjusted variables include age, menopausal years, sports physical activity, dietary energy, fat, isoflavones, BMI and equol producing status.

## Discussion

Our cross-sectional data among Hong Kong Chinese postmenopausal women demonstrated that equol/ODMA producers had a favourable cardiovascular risk profiles than non producers, suggesting that the ability to produce equol/ODMA might modulate cardiovascular risk factors.

### The Prevalence of Equol and O-DMA Phenotypes

The prevalence of equol and ODMA producers in present study were 53.2% and 60.9% respectively, similar to that previously reported frequency of equol and ODMA producers in Asians [22], [23], but differed with western populations with 20–30% equol producers and 80–90% ODMA producers in general [22]. An observational study also reported that Korean American women had a lower prevalence of *O-*DMA producers than Caucasian American women living in the same geographic area [24]. Thus, Asian women were more likely to be equol producers and less likely to be *O*-DMA producers than the western populations, suggesting that daidzein-metabolizing patterns differed between various ethnic groups [24]. The racial differences and genetic predisposition may influence the ability to metabolize daidzein [16]. Our results were also in line with previous observation that equol and *O-*DMA producing phenotypes are independent of each other [16]. Evidence from in vitro also supports that the bacteria that produce *O-*DMA are distinct from the bacteria that produce equol [25].

### Dietary Factors and Daidzein Metabolizing Phenotypes

Although diet has been reported to influence intestinal microbiota [26], dietary factors on daidzein metabolizing phenotypes are still inconclusive [24], [27], [28]. Some studies[27]–[29] have reported positive associations between equol production and intakes of soy, animal meat, green tea, or a low-fat high-carbohydrate diet while others not[24], [30]–[32]. In this study, although dietary information was collected using FFQ which would capture the regular food exposure, we observed little association between dietary nutrients and the daidzein-metabolizing phenotypes except for a lower total energy intake in equol producers. Hedlund et al. [33] reported that in Caucasians, individuals with a long-term high consumption of soy foods were more likely than low consumers to be equol producers. However, in our study, we did not detect a difference in habitual isoflavones intake between equol/ODMA producers and non producers. Given that the daidzein-metabolizing phenotypes appear to be stable over time, the ability to produce equol/ODMA may not be easily altered by dietary modification [30].

Since Asian women had regular soy consumption, a possible interaction may exist between phenotypes and soy consumption in relation to cardiovascular health. However our analysis stratifying equol and ODMA phenotypes observed most of non-significant associations between dietary isoflavones intake and cardiovscular risk factors, except a lower triglycerides level in equol producing group with high habitual isoflavones intake. The only significant finding among a number of comparisons might be treated with caution. The possible explanations for the mainly lack of association could be due to the inadequate statistical power from stratification analysis; women at high disease risk may modify their diet habit by intake of more healthy foods including soy; regular soy consumption in Chinese women of this age may have blunted the association; or factors not captured by our questionnaire are differentially related to cardiovascular markers and daidzein metabolizing patterns. Thus, the association of diet and daidzein metabolism should be further investigated in future studies.

### Cardiovascular Risk Factors and Daidzein Phenotypes

In the present study, we observed a favorable cardiovascular risk profiles (lower BP, lipids and hs-CRP etc.) in equol or ODMA producers in comparison to non-producers, suggesting a potential effect of equol/ODMA production on the prevention of cardiovascular diseases. Adjusting for potential confounders including age, dietary energy and lipids, sport PA and habitual isoflavones intake, did not alter the significance of the associations, implying the phenotypes may not influence cardiovascular risk through these factors. Consistent with previous observation [34], our study indicated that O-DMA production tent to demonstrate lower BMI and body fatness than non producers and the association was attenuated after adjustment for sports PA, suggesting that the favorable body composition in ODMA producers may somewhat relate to increased sports exercises. The study also showed that equol/ODMA producers were more likely to have more sports energy expenditure than non producers. Early studies indicated that physical activity has led to a faster gut transit time [35], [36], which could influence the metabolites produced by intestinal bacteria, including affecting the location and time available for metabolism of dietary compounds, and the composition and growth rate of the microbial community [37], and subsequently influence the disease risk.

### Mechanisms

The precise molecular mechanisms linking daidzein or its metabolite equol and O-DMA to cardiovascular health remained unclear. A likely explanation for the triglyceride reduction was that equol may activate PPARα or PPARδ, which leads to decreased triglyceride concentrations via increased fatty acid oxidation in liver or skeletal muscle etc. [38]. Equol has a greater antioxidant activity than genistein and daidzein, and can affect cell-mediated LDL modification by inhibition of NADPH oxidase activity [39].

In this study, we observed that the productions of equol and O-DMA were much variable among individuals following daidzein challenge. This observation suggested that a limited direct effect of circulating equol or ODMA and another mechanism may play a role on the cardiovascular risk and daidzein metabolizing phenotypes. Because intestinal bacteria metabolize estrogens and other steroid hormones [40], which are involved in the regulation of cardiovascular health, equol and ODMA production may represent intestinal bacterial profiles associated with hormonally-mediated factors independently of soy exposure. Additionally, urinary excretion of O-DMA in humans is a marker of harboring intestinal bacteria capable of C-ring cleavage. Bacterial C-ring cleavage reactions are relevant to other phytochemicals that may exert biological actions stronger than O-DMA; thus, the role of the phenotype may extend beyond daidzein metabolism [11]. Further research evaluating disease risk in relation to the equol/O-DMA phenotype from the perspective of intestinal microbial composition is warranted.

### Strengths

There are several strengths of our study. Firstly, compared with previous reports, our study had a relatively large sample size, included a comprehensive panel of cardiovascular risk factors and specifically conducted among postmenopausal women with pre-hypertension or untreated hypertension. In this population, the association was not influenced by chronic diseases or corresponding medication treatment. Secondly, equol and O-DMA status were defined by standardized methods by consecutive several days’ daidzein challenge to ensure sufficient exposure to daidzein and reveal the phenotypes accurately. Finally, we have shown for the first time that serum FFA was related with equol phenotypes, as previous report suggested that increased serum FFA levels are an important cause of obesity-associated insulin resistance and cardiovascular disease [41].

### Limitation

Our study has some limitations. First, it was a cross-sectional study and causal inferences cannot be made. Second, the parent study was a randomized trial, and the women were selected based on particular selection criteria, which may restrict the generalizability and reduce the variability in cardiovascular markers, thus decreasing the power to detect associations in this ancillary study. However, being pre- or hypertension is common among Hong Kong postmenopausal women as reported in Hong Kong 2003/2004 Population Health Survey that the prevalence of hypertension was 48.4% in women aged 55–64 [42] and midlife women (50–59 y) has the highest prevalence of prehypertension (42%) among all age groups [43]. Although we excluded patients with anti-hypertensive treatment, a local report revealed that only half of all hypertensive cases are diagnosed and half of those diagnosed are treated [44]. Thus, the selection criteria for prehypertension and untreated hypertension may have limited influence on the study generalizability. Finally, as previous study [29], ODMA producers in our study were defined as detectable urinary concentrations of ODMA. The ODMA cut-off has not yet been well-established and differed among studies.The higher or lower cut-off used could result in systematic shifting in classification. Thus, we conducted further analyses using various cut-offs such as 170 nmol/l [24] or 339 nmol/l [45] or ODMA/creatinine ratio of 0.018 [23] for defining ODMA status, however, it made little misclassification and influence on the results.

## Conclusion

Our cross-sectional data suggested that equol and O-DMA producers than non-producers had better cardiovascular health profiles in Chinese postmenopausal women with prehypertension or untreated hypertension. The associations were independent of dietary or other lifestyle factors. Further studies characterizing the associations of intestinal bacterial profiles with cardiovascular markers are warranted.
